# Regulation and mechanisms of action of RNA helicases

**DOI:** 10.1080/15476286.2024.2415801

**Published:** 2024-10-22

**Authors:** Nina Lang, Pravin Kumar Ankush Jagtap, Janosch Hennig

**Affiliations:** a Chair of Biochemistry IV, Biophysical Chemistry, University of Bayreuth, Bayreuth, Germany; b Molecular Systems Biology Unit, EMBL Heidelberg, Heidelberg, Germany

**Keywords:** RNA helicases, RNA remodelling, RNA unwinding, autoregulation, auxiliary domains, DHX9

## Abstract

RNA helicases are an evolutionary conserved class of nucleoside triphosphate dependent enzymes found in all kingdoms of life. Their cellular functions range from transcription regulation up to maintaining genomic stability and viral defence. As dysregulation of RNA helicases has been shown to be involved in several cancers and various diseases, RNA helicases are potential therapeutic targets. However, for selective targeting of a specific RNA helicase, it is crucial to develop a detailed understanding about its dynamics and regulation on a molecular and structural level. Deciphering unique features of specific RNA helicases is of fundamental importance not only for future drug development but also to deepen our understanding of RNA helicase regulation and function in cellular processes. In this review, we discuss recent insights into regulation mechanisms of RNA helicases and highlight models which demonstrate the interplay between helicase structure and their functions.

## Introduction

RNA helicases are conserved enzymes utilizing nucleoside triphosphates (NTPs) to either bind, remodel or unwind nucleic acids, ribonucleoprotein (RNP) complexes, or both [1]. They are ubiquitous across bacteria, archaea, eukaryotes, and several viruses underscoring their significance in various biological systems [2]. The classic definition of helicases describes them as enzymes which possess unwinding activity. However, the mechanisms and functions of helicases are much more diverse as some helicases can clamp onto nucleic acids or even promote strand annealing [3–7]. In eukaryotes, helicases participate in almost every RNA or DNA related process in the cell such as transcription, translation, pre-mRNA splicing, rRNA processing, or in resolving G-quadruplexes [8–11]. Therefore, it is not surprising that appropriate function and regulation of RNA helicases is necessary for cellular homeostasis and viability. Furthermore, some viruses have been shown to hijack cellular RNA helicases to promote and facilitate viral gene expression, replication and packing of emerging viral particles [12,13]. Due to crucial regulatory roles of RNA helicases in the cell, their dysregulation or altered expression has been shown to result in several diseases including cancer, neurological disorders, and developmental defects [9,14].

Based on structural and mechanistic features as well as characteristic and conserved sequence motifs, helicases have been classified into six superfamilies (SF) [15]. SF1 and SF2 include monomeric helicases to which all eukaryotic RNA helicases belong. SF3 to 6 encompass ring-forming helicases, which mostly form hexameric complexes that assemble around RNA. The common characteristic of SF1 and SF2 helicases is the conserved helicase core consisting of two similar recombinase A (RecA)-like domains, while the subdivision of these superfamilies is based on characteristic sequence motifs involved in ATP binding and hydrolysis as well as in RNA binding. DEAD box and DEAH/RHA helicases are the largest families of RNA helicases in humans. They share a conserved helicase core consisting of two RecA domains, which include at least 12 of these characteristic sequence motifs [15–19]. The conserved helicase core is often flanked by N- and C-terminal auxiliary domains or extensions, which have been shown to mediate substrate specificity or possess regulatory functions [15,20–22]. These include structured domains such as double-stranded RNA binding domains (dsRBDs), zinc-fingers, RNA recognition motifs (RRMs), oligosaccharide-binding folds or caspase activation and recruitment domains (CARDs) [15,20,21,23–25], and intrinsically disordered regions (IDRs) [26,27].

Although cellular functions of various RNA helicases are well-known, how they fulfill their cellular tasks and how they are regulated at a molecular level remains mostly unclear. Therefore, unravelling the regulation and mechanism of RNA helicases will not only deepen our understanding of fundamental cellular processes but will also provide the basis for development of highly specific inhibitors. Identifying unique features within helicases is of fundamental importance to precisely target specific helicases without affecting orthologous helicase family members. By employing structural biology methods, different conformational states of several RNA helicases could be captured, which deepen our understanding about their molecular dynamics and regulation mechanisms. As the helicase core is conserved, unique auxiliary domains and features have been shown to play major functional and regulatory roles. This review aims to highlight recent research demonstrating intricate mechanisms by which some of these RNA helicases operate.

## Diversity of RNA helicase mechanisms

Although, RNA helicases are best known for unwinding RNA duplexes, their molecular mechanisms have evolved to a higher diversity. These functions and mechanisms of RNA helicases have been reviewed extensively [8,9,28–32]. RNA helicases mediate duplex unwinding using two basic mechanisms characterized by either local duplex unwinding or processive translocation along RNA. Due to a linker region between the two conserved RecA-like domains, they can change their relative orientation to each other, enabling different conformational states. Opening and closure of these two domains are characteristic of all eukaryotic RNA helicases [33]. Furthermore, auxiliary domains have been shown to mediate crucial conformational rearrangements. Generally, in the absence of ATP and RNA, RNA helicases adopt an open conformation representing an inactive state, while binding of both, ATP and RNA, induces a closed conformation resembling a compact active state. Due to ATP binding and the resulting inter-domain interactions in the closed state, ATPase activity is stimulated and the bound RNA is remodelled or unwound [34]. Thus, repetition of opening and closing of the two RecA-like domains enables several subsequent helicase cycles [35].

**Figure 1.**
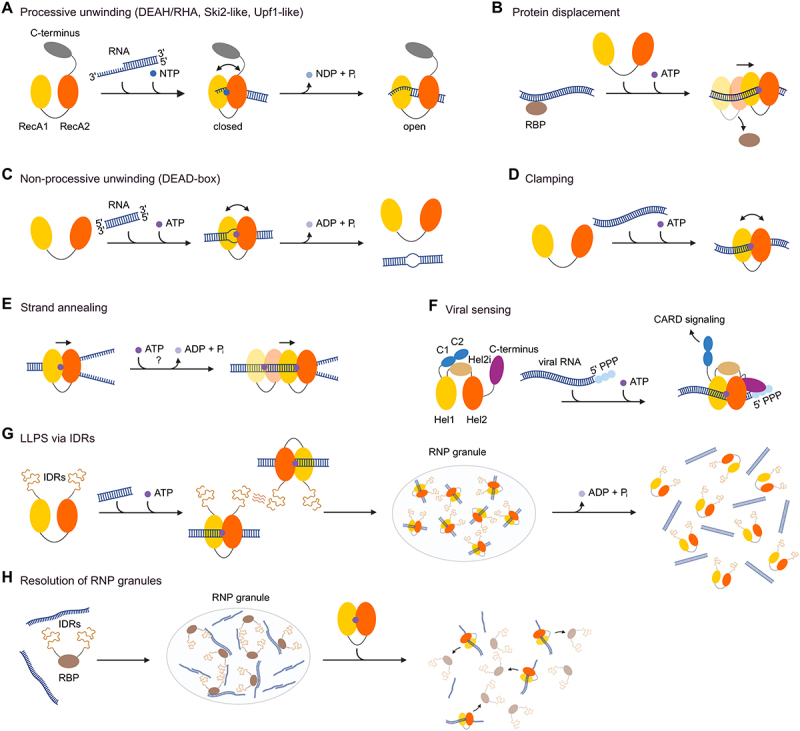
Mechanisms of RNA helicases adapted from Bohnsack et al.^9^

DEAH/RHA, Ski2-like and Upf1-like helicases typically translocate along ssRNA while unwinding long stretches of RNA and require a 3’ single stranded overhang to allow loading onto RNA (Figure 1A). However, some helicases have been shown to load independently onto nucleic acids. Furthermore, binding of specific protein cofactors has been shown to modulate unwinding processivity [36]. Translocation along RNA without any strand separation can dissociate bound proteins from RNA, thereby regulating protein:RNA interactions (Figure 1B) [37].

(A) Processive RNA unwinding of DEAH/RHA, Ski2-like and Upf1-like helicases. (B) While translocating along RNA some helicases are known to displace RNA-binding proteins (RBPs) from RNA/DNA. (C) Non-processive RNA unwinding mechanism of DEAD-box helicases with local strand unwinding. (D) If ATPase activity is blocked, RNA helicases can clamp onto their substrate and serve as a assembly platform for additional proteins. (E) Some RNA helicases have been proposed to facilitate strand annealing. The question mark indicates that the importance of ATP-hydrolysis of this process is not fully understood. (F) RIG-I like helicases are capable of sensing viral RNA. RNA-binding induces a conformational change, which enables auxiliary domains to specifically recognize unique features of viral RNA. Subsequently, innate immune responses are initiated. (G) RNA helicases containing IDRs have been shown to be involved in liquid-liquid phase separation (LLPS). ATP-dependent binding and multivalent interactions formed by IDRs can promote the formation of ribonucleoprotein (RNP) granules. ATP hydrolysis often triggers dissociation of the RNA helicase from the RNA, thus, leading to disassembly of the RNP granule. IDR: intrinsically disordered region. (H) RNA helicases can antagonize RNP granule formation by resolving RNA-protein assemblies. The two conserved RecA helicase domains are shown in yellow and orange.

Non-processive RNA unwinding is widespread among DEAD-box helicases, which are the largest helicase family containing more than 30 members in humans (Figure 1C) [38]. The conserved sequence motif Asp-Glu-Ala-Asp (DEAD) at the active site gives this helicase family its name. Furthermore, the unique Q-motif at the ATP binding site mediates ATP-specific binding. It consists of 9 amino acids with a conserved glutamine residue, which specifically hydrogen bonds with the N6 and N7 position of the adenine. In addition, a conserved phenylalanine, 17 amino acids upstream of the glutamine stacks onto this adenine [17]. Binding of ATP and RNA promotes the conformational transition from an open to a closed state leading to the formation of a functional ATP-hydrolysis site [39]. As non-processive RNA helicases DEAD-box helicases mediate strand separation instead of unwinding long stretches of RNA. This activity only requires ATP binding, while ATP hydrolysis promotes dissociation of the helicase from the RNA [40,41].

In both DEAD-box and DEAH/RHA families, the majority of determined RNA bound helicase structures revealed no base specific RNA binding within the conserved motives of the RecA-like domains but rather non-specific interactions with the backbone and the 2’ OH groups [39]. Substrate specificity can be established by recruitment to target RNAs or RNPs mediated in *cis* by auxiliary domains [21,42,43]. A prominent example is the RNA helicase YTHDC2, which is targeted to m6A-containing RNAs through its YT521 homology (YTH) domain [44,45] or retinoic acid inducible gene I (RIG-I), which specifically recognizes viral dsRNA carrying 5’ di- or triphosphates with its Hel2i and C-terminal domains [21]. Furthermore, interaction partners can recruit the RNA helicase to its RNA substrate in *trans*. For example the complex of DHX15 and NLRP6 acts as a viral RNA sensor and induces interferon-stimulated genes [46]. The interaction with NLRP6 stabilizes the interaction between DHX15 and MAVS and is therefore essential to activate downstream interferon-stimulated genes [46–48]. Although both, DHX15 and NLRP6, bind single stranded viral RNA, DHX15 seems to act as the primary RNA sensor [46]. However, the interaction between DHX15 and NLRP6 in addition triggers NLRP6 inflammasome assembly resulting in the activation of an immune response by secretion of IL-18 [48]. Recent findings reveal that NLRP6 inflammasome activation is mediated by liquid – liquid phase separation, with DHX15 forming condensates together with NLRP6 and RNA [49].

In addition to the well-known unwinding activity of RNA helicases, several other mechanisms have evolved. For example, ATP-dependent RNA clamping has been observed for some helicases [50]. When ATP hydrolysis is inhibited, the helicase remains permanently bound to RNA in a state called clamping (Figure 1D). A prominent example is eIF4A-III, which is characterized by a minimal helicase core structure comprising solely RecA1 and RecA2 domains. Instead of unwinding RNA, eIF4A-III clamps on RNA as it displays the core of the exon junction complex (EJC) providing a nucleation centre to assemble the complex. This clamping is achieved by the stabilization of the ADP-Pi-bound state, which is accomplished through the interaction of the MAGOH-Y14 dimer with eIF4A-III [3,4,51].

Several RNA helicases have also been reported to harbour chaperone activity and thus promote ATP-independent strand annealing (Figure 1E) [5,52–55]. By catalysing both RNA unwinding and annealing, these helicases can facilitate the rearrangement of RNA secondary structures. In addition to the above two mechanisms, members of the RIG-I like RNA helicase family do not unwind RNA duplexes. Instead, they bind dsRNA in their RNA binding tunnel and sense specific duplex ends. Subsequently, they initiate innate immune responses with their auxiliary domains (Figure 1F) [56,57].

Liquid-liquid Phase Separation (LLPS) has recently emerged as a fundamental molecular mechanism enabling cells to rapidly and reversibly form dynamic membraneless organelles including nucleoli or RNA granules such as processing bodies (P-bodies) and stress granules [58]. LLPS is a biological phenomenon in which certain proteins and nucleic acids undergo phase transition from a soluble state into a condensed liquid-like phase. This process enables the formation of biomolecular condensates in which specific molecules are highly concentrated and thereby facilitate localized biochemical reactions and cellular processes. Aberrant condensate formation has been associated with various diseases such as cancer and neurodegeneration [58–60]. Since LLPS relies among others on protein-protein, RNA-protein and RNA-RNA interactions, it is not surprising that RNA helicases have been reported to be important regulators of such biomolecular condensates either by directly participating in their formation or by modulating their dynamics and composition (Figures 1G, H) [32,57–61]. These RNA helicases, such as DHX9 and eIF4A, are known to interact with RNA and other proteins involved in LLPS. Stress granules formed in daughter cells upon UV-induced crosslinking damage in mother cells contain DHX9, which selectively enrich damaged intron RNA but not DNA. By modulating dsRNA abundance in the cell these DHX9 stress granules promote cell survival and induce dsRNA-related immune responses. Thus, DHX9 stress granules protect the daughter cells from parental RNA crosslinking damage [62]. However, to date there is no structural basis of DHX9s molecular grammar during LLPS. It has been shown that intrinsically disordered regions (IDRs), which are also present in DHX9, can promote LLPS (Figure 1G) . DEAD-box helicases have been shown to promote phase separation in their ATP-bound form and in presence of RNA and control the RNA flux into and out of membraneless organelles [32,63]. ATP-hydrolysis triggers release of RNA and in consequence leads to the disassembly of the biomolecular condensate. (Figure 1H) [32,64].

In addition to promoting LLPS, certain RNA helicases can also negatively influence LLPS. The DEAD-box helicase eIF4A has been reported to disrupt RNA-RNA interactions which are crucial during stress granule formation and thus induces RNA decondensation. In this process ATP-dependent binding of eIF4A is sufficient to limit RNA-dependent stress granule formation [61]. However, ATP hydrolysis may increase the negative effect of eIF4A on stress granule formation by allowing multiple cycles of RNA binding.

## Regulation of SF2 RNA helicases

RNA helicases participate in various RNA-related cellular processes such as transcription, translation, pre-mRNA splicing, rRNA processing or RNA degradation. Due to the complex cellular environment precise regulation of helicase activity is therefore crucial for proper timing and fidelity of these processes. Precisely regulated helicase function is not only critical for embryonic development but also for maintaining cellular homeostasis. As the conserved helicase core displays a basal ATP-dependent helicase activity, it needs to be precisely stimulated or inhibited in a spatio-temporal manner.

Most of SF1 and SF2 RNA helicases contain N- and C-terminal auxiliary domains, which contribute to the functional diversity of RNA helicases by introducing substrate specificity or regulatory moieties. These N- and C-terminal extensions recently have been shown to carry out important regulatory functions. Furthermore, self-association of some RNA helicases has been shown to further regulate helicase activity. Binding of protein cofactors in *trans* provides another layer of RNA helicase regulation as they can either enhance or inhibit helicase activity by inducing structural rearrangements. Moreover, additional specificity can be introduced by binding of protein cofactors. Depending on the cellular context helicase activity can be regulated by binding of a spatially or temporarily specific subset of available protein cofactors. Next to individual cofactors, two main evolutionary conserved domains, namely G-patch and MIF4G (middle domain of eukaryotic initiation factor 4 G) domains, have evolved and display key roles for modulating RNA helicase activity [30]. Most of them act as conformational regulators by inducing structural rearrangements, which either promote or inhibit ATPase and helicase activity.

Recent structural and mechanistic insights into modes of action of a growing number of RNA helicases reveals remarkable insights into their mechanisms and intrinsic regulation, which will be discussed in the following.

### Autoinhibition of RNA helicases in the absence of substrates

**Figure 2.**
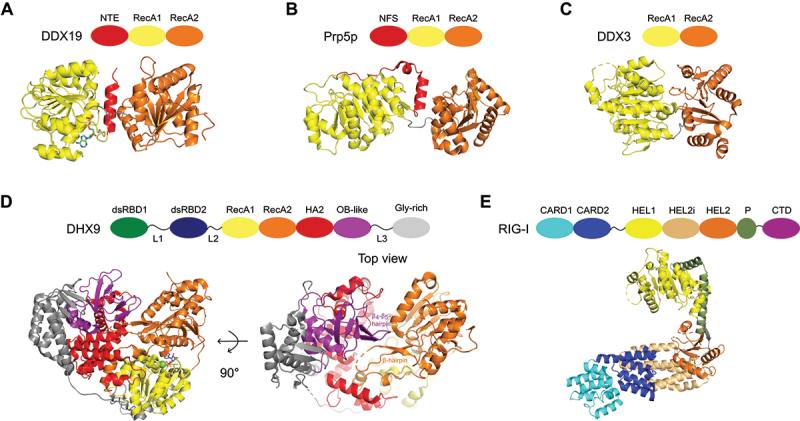
Autoinhibitory mechanisms of RNA helicases in the absence of RNA and ATP.

Multiple RNA helicases are in an autoinhibited state in the absence of their substrate, in which either the RNA binding site is inaccessible or ATP hydrolysis is prevented [20,25,30,57]. Structures of several autoinhibited conformational states have been reported. One mechanism is the stabilization of the spatial separation between the two RecA-like domains in the absence of RNA and ATP. In DDX19 an N-terminal α-helix inserts between the two RecA-like domains which prevents ATP hydrolysis in the absence of RNA (Figure 2A) [65]. Prp5p evolved an analogous mechanism with an inhibitory α-helical insertion (Figure 2B) [66]. In DDX3 interdomain interactions between RecA1 and RecA2 stabilize a partially closed conformation (Figure 2C). As this interface in addition overlaps with the RNA binding surface, this conformation is inhibitory to RNA duplex unwinding [67]. Furthermore, the RNA tunnel can be occluded by several structural elements as it has been shown for MLE and its human ortholog DHX9 [20,25]. As part of the DEAH/RHA RNA helicase family MLE’s and DHX9’s RNA binding tunnel is formed by a trefoil arrangement of RecA1, RecA2 domains and the HA2-OB-L3 module [20,25]. In the absence of RNA, the RNA binding tunnel of DHX9 is occupied by the β4-β5 loop of the OB domain and the α6-α7 loop of HA2 domain, similar to MLE (Figure 2D) [25]. In addition, αB-helix of RecA2 further occludes the RNA entry site in MLE. However, the density of αB-helix of RecA2 is missing in the DHX9 crystal structure suggesting that the inhibitory mechanism might be slightly different in the two orthologs [20].

(A) Autoinhibition of DDX19 by insertion of an N-terminal α-helix between the RecA-like domains (PDB: 3EWS). The N-terminal α-helix inserts into the central cleft, thereby preventing ATP-hydrolysis. NTE: N-terminal extension, RecA1 and RecA2: helicase domains. (B) A similar insertion of an N-terminal α-helix is observed for Prp5p in the absence of substrates (PDB: 4LK2). NFS: N-terminal flanking sequence. (C) A partially closed conformation in the absence of RNA is observed in DDX3. This conformation is inhibitory to RNA duplex unwinding. (D) In the DEAH/RHA RNA helicase DHX9 the RNA binding tunnel is occluded by several structural elements such as the β-hairpin of RecA2 and the OB-like β4-β5-strands in the absence of RNA (PDB: 8SZP). A similar mechanism is observed for its *Drosophila melanogaster* ortholog MLE. dsRBD: double-stranded RNA binding domain, RecA: helicase domain, HA2: helicase associated domain, OB-like: oligosaccharide binding-like domain. (E) Autoinhibition of RIG-I in the absence of RNA. The CARDs are sequestered to the helicase core and subsequently are not susceptible for immune signalling (PDB: 4A2W). CARD: caspase activation and recruitment domain, HEL1 and HEL2: helicase domains, HEL2i: helicase insertion domain, P: pincer domain, CTD: C-terminal domain. The colour-code is according to the domain architecture shown above.

Another mechanism of RNA helicase regulation is the inhibition of downstream signalling in the absence of RNA and ATP. The most prominent example is RIG-I, which adopts an autoinhibitory conformation in the absence of RNA [24,68]. In this state its N-terminal caspase activation and recruitment domains (CARDs) are stably bound to the helicase and thus are not susceptible for immune signalling (Figure 2E). When viral RNA is present, it binds to the C-terminal domain (CTD) of RIG-I, leading to a conformational change that releases the CARDs. After ubiquitination by TRIM25 or Riplet, the exposed CARDs can interact with the mitochondrial antiviral-signalling protein (MAVS) which triggers downstream signalling and the induction of type I interferons and other antiviral responses [69–71].

In summary, autoinhibited states of RNA helicases are crucial for ensuring that these enzymes are only active when needed. Autoinhibition allows RNA helicases to be highly responsive to specific signals such as viral RNA or other substrates. By maintaining an autoinhibited state in the absence of their target RNA, RNA helicases avoid unnecessary activation which prevents for example ATP hydrolysis in the absence of cognate RNA or unwarranted immune responses that could lead to inflammation or autoimmunity. In addition, cellular homeostasis is maintained as continuous activation of helicases could disrupt normal cellular processes, leading to detrimental effects on cell function and health. Furthermore, autoinhibition ensures that ATP is not wasted on unwarranted activity, conserving cellular energy for processes that are immediately necessary. Interestingly, AMP binding can inhibit RNA unwinding in some RNA helicases, indicating an additional regulatory sensor function of the helicase regarding the energy charge of the cell [72].

### Essential roles of auxiliary domains in RNA helicase regulation

**Figure 3.**
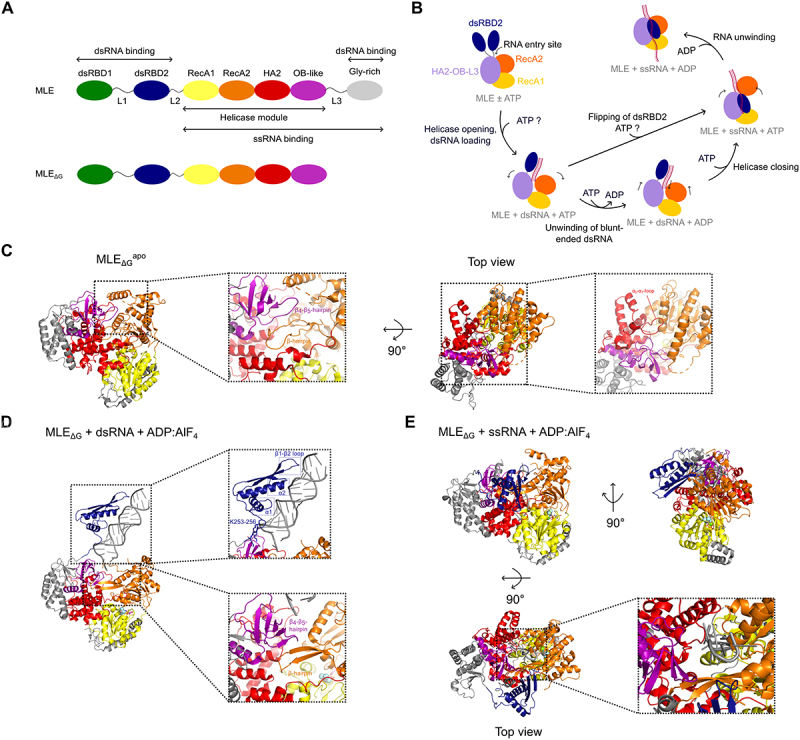
Structural basis of autoregulation of *Drosophila melanogaster* DEAH/RHA helicase MLE.

Recent studies highlighted the importance of auxiliary domains in regulating helicase activity by recognizing specific substrates, inducing crucial conformational changes which alter the activity and preference for different RNA substrates or releasing the helicase from an autoinhibited state. A recent study demonstrated how auxiliary domains regulate the helicase cycle [20]. The *Drosophila melanogaster* DEAH/RHA helicase maleless (MLE) is well-known in the context of the male-specific-lethal (MSL) dosage compensation complex (DCC), which mediates two-fold hypertranscription of the single X-chromosome in male flies [73–75]. The essential role of MLE in this context is the remodelling of long non-coding RNA roX2 into an alternative conformation which enables subsequent assembly of the MSL complex [76]. MLE’s domain architecture is characterized by two N-terminal double-stranded RNA binding domains (dsRBDs), followed by the helicase core consisting of RecA1, RecA2, HA2 and OB-like domains and a C-terminal glycine-rich region (Figure 3A) [77].

(A) Domain architecture of MLE. Single-stranded and double-stranded RNA-binding regions are indicated. The construct used for all cryo-EM studies (MLE_ΔG_) lacks the C-terminal intrinsically disordered Gly-rich region. dsRBD: double-stranded RNA binding domain, RecA: helicase domain, HA2: helicase associated domain, OB-like: oligosaccharide binding-like domain. (B) Model for MLE cis-regulation of RNA-binding and helicase activity with dsRBD2 acting as a cis-regulatory unit. Question marks indicate that the order of RNA and ATP binding is not known. (C) MLE_ΔG_ in the apo state (PDB: 8B9L). In this state the RNA binding tunnel is occluded by the β-hairpin of RecA2 and the OB-like β4-β5-strands. The top view shows that α6-α7-loop of HA2 is preventing access of ssRNA into the tunnel. (D) MLE_ΔG_ bound to dsRNA and ADP:AlF_4_ (PDB: 8B9K). dsRBD2 contacts dsRNA with the three conserved RNA-binding regions. K253-256 contact and guide the RNA to the tunnel. The RNA binding tunnel is still occluded by the β-hairpin of RecA2 and the OB-like β4-β5-strands. (E) MLE_ΔG_ bound to ssRNA and ADP:AlF_4_ (PDB: 8B9I). dsRBD2 is bound to the helicase core, inducing structural rearrangements in the helicase tunnel. ssRNA is accommodated in the tunnel. The colour-coding is according to (A).

Prior functional studies of MLE showed that the auxiliary dsRBD2 domain but not dsRBD1 is indispensable for helicase activity and localization to the X chromosome *in vivo* indicating a fundamental role of dsRBD2 for proper helicase function [78]. Structural studies of the isolated tandem dsRBDs revealed RNA binding capacity for both dsRBD domains *in vitro* with dsRBD2 having a 14-fold higher affinity to dsRNA than dsRBD1 [23]. Recent work by Jagtap et al. revealed a model for the RNA unwinding process of MLE by capturing it in different conformational states during the RNA binding and unwinding process using cryo-EM [20]. A previously published crystal structure of the MLE_core_, lacking dsRBD1 and the C-terminal Gly-rich region, already showed that dsRBD2 interacts with the helicase module in the ssRNA- and ATP-bound state resembling a closed conformation of MLE. This interaction is established by electrostatic and hydrophobic contacts of dsRBD2 with the OB-like domain, HA2 and the β-hairpin of RecA2 [77]. Interestingly, dsRBD2 contains a specific N-terminal α0 helix which also binds to the OB-like fold. The functional importance of the dsRBD2-helicase module interface is further supported by *in vitro* and *in vivo* experiments, in which the conformation of dsRBD2 is crucial for proper RNA binding, helicase activity and localization to the X-chromosome territory [20]. The structural studies supported by cryo-EM structures of MLE_ΔG_ (lacking the C-terminal Gly-rich region) captured several states during the helicase cycle of MLE, allowing to establish a functional model of its helicase cycle (Figure 3B) [20]. In the absence of substrate, MLE_ΔG_ adopts a compact structure consisting of three lobes corresponding to RecA1, RecA2 and the HA2-OB-L3 module (Figure 3C). The two N-terminal dsRBD domains have no fixed orientation with respect to the helicase core domain and remain mobile in this state and thus are not visible in the cryo-EM density. The β-hairpin of RecA2 and the OB-like β_4_-β_5_-strands are moved inwards, which occludes the ssRNA-binding tunnel (Figure 3C). A similar inactive state has also been recently published of MLEs human ortholog DHX9^25^. Together with the high sequence and structure conservation between MLE and DHX9, these results indicate that DHX9 might have a similar mechanism as MLE. Only upon binding of dsRNA, opening of the helicase module is observed in MLE while binding of ATP alone does not induce major conformational changes. This is in contrast to other RNA helicases such as Prp43 in which ATP-binding induces a conformational change which opens the ssRNA-binding tunnel [79].

The cryo-EM structure of MLE_ΔG_ in complex with double stranded RNA substrate and ATP-analogue captures dsRBD2 bound to dsRNA while dsRBD1 remains invisible indicating high mobility (Figure 3D). The structure shows that dsRBD2 contacts the dsRNA with the three conserved RNA-binding regions consisting of the loop between β1-β2, α1 helix and α2 helix typical for dsRBDs [80]. dsRBD2 positions dsRNA directly at the entry of the ssRNA binding tunnel. Conserved lysines (K253-K256) in the linker between dsRBD2 and helicase core stabilize correct positioning of the RNA. Upon ATP-hydrolysis 3’ to 5’ translocation is initiated with RecA2 and the HA2-OB-L3 domains moving towards the 5’-end of the RNA while the 3’-end is pulled into the RNA tunnel. To allow ssRNA to completely enter the tunnel further conformational changes need to occur with dsRBD2 releasing the dsRNA and flipping back onto the helicase core which triggers the RecA2 domain and the HA2-OB-L3 module to move inwards thereby closing the RNA binding tunnel around the ssRNA (Figure 3E). In this state dsRBD2 forms several hydrophobic and electrostatic contacts with the helicase core [77].

The change between the so-called flipped-in and flipped-out state of dsRBD2, either being bound to the helicase core or bound to dsRNA, thus modulates MLE’s RNA preference. Therefore, dsRBD2 can be described as a autoregulatory unit. This example demonstrates that auxiliary domains can facilitate not only substrate recruitment but also regulate structural rearrangements and alter substrate preferences during the RNA unwinding process. Furthermore, this example shows that studying orthologs of essential human RNA helicases can help to identify unique characteristics that may be conserved among orthologs. In this specific case, the mechanism of MLE may provide hints on how its human ortholog DHX9 is regulated. This is of particular interest since overexpression and upregulated activity of DHX9 has been observed in several cancer types [81–85]. Notably, tumour cells exhibit a higher dependency on DHX9, while systemic knockdown is well-tolerated in adult mice, making DHX9 a potential therapeutic target [86]. Despite the fact that cellular functions of DHX9 are well-known, there is only limited knowledge and structural information about the underlying molecular mechanism of DHX9. Knowing the regulatory mechanism of DHX9 on a detailed molecular level may provide the basis for future drug development to specifically target DHX9 in human cells. Being able to specifically target one RNA helicase is crucial to maintain cellular homeostasis. As the helicase core including its ATP-binding site is conserved among RNA helicases, the unique N- and C-terminal auxiliary domains display promising target sites. Therefore, a detailed structural and mechanistic understanding of how DHX9 is regulated is crucial to precisely interfere with this process and regulate its activity.

**Figure 4.**
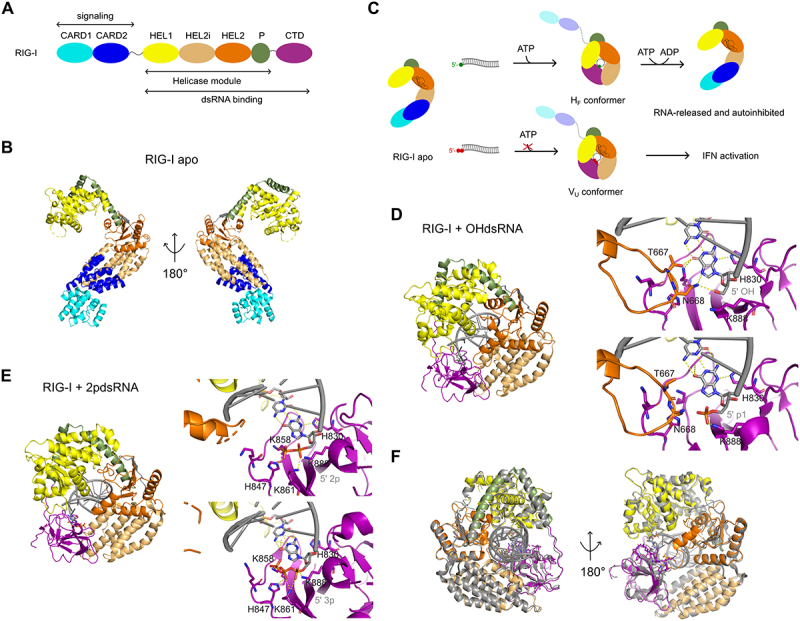
Structural basis of the autoregulation and specificity of RIG-I.

Another prominent example of auxiliary domain mediated regulation of the downstream RNA helicase function and substrate specificity is illustrated by RIG-I. As part of the RIG-I like helicase family, RIG-I is a well-known immune receptor which detects viral genomic RNA carrying a 5’-diphosphate or triphosphate moiety [87–89]. Rapid and effective detection of small traces of viral RNA upon infection is crucial to initiate innate immune responses. However, at the same time RIG-I needs to faithfully discriminate against host RNA which is highly abundant in the cytoplasm. The domain architecture of RIG-I diverges from that of a typical RNA helicase (Figure 4A). RIG-I contains several non-conserved features such as a regulatory C-terminal domain (CTD) and the two N-terminal caspase-activation and recruitment domains (CARDs), which are well-known to interact with the mitochondrial antiviral-signalling protein (MAVS) and trigger a downstream signalling cascade stimulating transcription of interferons [21,24,57]. Furthermore, another unique feature of RIG-I like receptors is called the Pincer domain, a V-shaped helical structural element located C-terminal of the helicase core. It is critical for its function as it connects and coordinates Hel1, Hel2 and the CTD and directly contacts RNA [88,90]. Notably, RIG-I binds dsRNA in its RNA tunnel, in contrast to most SF2 helicases, which accommodate ssRNA. Another unique structural feature is Hel2i, which is an α-helical bundle inserted between Hel1 and Hel2 and serves two significant functions. On the one hand Hel2i enables dsRNA binding by engaging with 2’-hydroxyl groups on both strands of dsRNA. On the other hand, Hel2i sequesters the CARDs on the surface of RIG-I in the absence of RNA and ATP representing an autoinhibited state (Figure 4B). In the case of RIG-I closure of the helicase core upon RNA binding is not coupled to duplex unwinding but rather activates downstream signalling through the CARDs as the conformational change exposes the CARDs [68].

(A) Domain architecture of RIG-I. Double-stranded RNA-binding and signalling regions are indicated. CARD: caspase activation and recruitment domain, HEL1 and HEL2: helicase domains, HEL2i: helicase insertion domain, P: pincer domain, CTD: C-terminal domain. (B) *Anas platyrhynchos* RIG-I in the absence of substrate (PDB: 4A2W). (C) Model for substrate activation and triggering of viral signalling of RIG-I. (D) *Homo sapiens* RIG-I bound to cellular OHdsRNA (PDB: 7TO0). The zoomed in views show specific contacts to OHdsRNA (top, PDB: 7TO0) and p1dsRNA (bottom, PDB: 7TNZ). (E) *Homo sapiens* RIG-I bound to viral p2dsRNA (7TNY). The zoomed in views show specific contacts to p2dsRNA (top, PDB: 7TNY) and p3dsRNA (bottom, PDB: 7TNX). (F) Overlay of the p3dsRNA-bound state (grey) and the OHdsRNA bound state (colour) demonstrating the destabilization of Hel2 upon binding of viral RNA. The OHdsRNA-bound state shows a well-folded Hel2 domain (orange), while it is destabilized in the p3dsRNA-bound state. The colour-coding is according to (A).

How RIG-I can distinguish viral RNA carrying 5’-diphosphates or 5’-triphosphates from cellular RNA was unknown for a long time. To specifically recognize such a unique molecular feature of viral RNA which differs from host RNA in as little as one phosphate group, remarkable specificity and precise structural arrangements need to be carried out by RIG-I. Wang and Pyle recently presented the first RNA bound RIG-I structures in the full-length context revealing the basis of this impressive selective recognition of viral RNA and propose a functional model (Figure 4C) [88]. Despite the RNA-binding mode of the dsRNA in the RNA tunnel being similar in all structures, the CTD and Hel2 undergo different structural changes dependent on either host or viral RNA binding. The cryo-EM structures revealed the presence of two distinct conformational states, the host-folded (H_F_) and the viral unfolded (V_U_) conformer, that distinguish viral from host RNA and alters downstream signalling behaviour of RIG-I (Figure 4D). A surprising observation was regardless of which RNA is bound, in any case the CARDs are exposed and in principle are available for downstream signalling even in the presence of host RNA. However, RIG-I does not trigger interferon responses in the cell in the absence of viral RNA. The H_F_ conformer was captured in the presence of host mimicking RNAs bearing either a 5’ monophosphate or 5’ OH. Interestingly, the conserved helicase motifs of Hel2 are well folded in contrast to the apo structure in which Hel2 is relatively unfolded [24,88]. Maintaining the structural integrity of the motif IVa is critical in RIG-I as it contains unusual domain insertions which reduce their stability, thus displaying a critical function in regulating the activity of RIG-I. Another critical feature is the Hel_TNT_ insertion in Hel2, which contacts the CTD and is involved in the recognition of 5’ ends of host RNAs. The critical residue N668 in this motif interacts with the 5’-OH group of host RNA and H847 of the CTD, while in the presence of 5’-monophosphorylated RNA (p1dsRNA) it clashed with the α-phosphate, thus N668 adopts a different conformation. Instead, the CTD (K888) interacts with the α-phosphate of the RNA (Figure 4D). Importantly, in both cases Hel2 is well-folded enabling ATP-hydrolysis and translocation along dsRNA which leads to rapid dissociation of RIG-I from host RNA. Distinct structural changes are observed in the V_U_ conformer captured by RIG-I bound to 5’-diphosphorylated RNA (p2dsRNA) and 5’-triphosphorylated RNA (p3dsRNA) (Figure 4E). The structures show that the primary determinant for the recognition of viral RNA is the β-phosphate as the CTD contacts α- and β-phosphate but not the γ-phosphate. The 5’-β-phosphate prevents docking of the Hel_TNT_ motif which leads to unfolding of Hel2 and subsequent destabilization of the Hel2 domain. The overlay of both conformers (H_F_ and V_U_) nicely shows that the position of the β-phosphate would clash with Hel_TNT_, indicating that viral RNA destabilizes the structural integrity of Hel2 (Figure 4F). Consequently, motif IVa is not stabilized in the viral RNA bound state and the ATP binding motifs are not correctly positioned, thus RIG-I cannot hydrolyse ATP when bound to viral RNA. This is consistent with previous results as it has been shown that RIG-I clamps on dsRNA with 5’-triphosphates, but not on cellular dsRNA with 5’- OH [91]. Furthermore, the CTD forms an extensive interaction interface with Hel2i, which is expected to completely block reassociation of the CARDs preventing the formation of the autoinhibited state. This results in a robust exposure of the CARDs leading to downstream signalling and triggering immune response. The conformational state of RIG-I dependent on either host or viral RNA results in either the formation of a functional or a non-functional ATP-binding site, respectively. Thus, ATP binding displays a proof-reading mechanism which ensures that signalling is only possible when RIG-I is clamped on viral RNA. In contrast ATP hydrolysis stimulates translocation of RIG-I along host dsRNA, which leads to rapid dissociation of RIG-I, preventing downstream signalling.

Apart from intrinsic regulation, post-translational modifications have also been shown to regulate helicase activity. RIG-I serves as an ideal example as it was shown that acetylation prevents RIG-I oligomerization. During viral infection, RIG-I acetylation is removed by HDAC6 enabling RIG-I signalling [92]. Interestingly, viral helicases such as the NS3 helicase of flaviviruses have been reported to get acetylated leading to their regulation of the helicase activity [93]. These findings highlight the importance of studying RNA helicases from other organisms such as viruses or pathogens thereby helping to reveal common features of helicase regulations conserved across species.

Moreover, the interplay between virus and host might also represent an important helicase regulatory mechanism. For RIG-I, it has been shown that a deaminase of herpes simplex virus 1 (HSV-1) deamidates the side chain amides of two asparagine residues in the Hel2i domain of RIG-I. As a result, this deamidation impairs recognition of viral RNA subsequently abrogating RIG-I dependent immune signalling [94]. These findings suggest that posttranslational modifications such as acetylation may represent a broader mechanism of helicase regulation. As *in vitro* studies of RNA helicase regulation are mostly based on purified proteins, the important posttranslational modifications present *in vivo* are often ignored.

Unique features of RNA helicases, mostly introduced by non-conserved auxiliary domains, play crucial roles in regulating their activity as it has been shown for MLE and RIG-I recently. Structural studies of these two helicases demonstrate the impact of cryo-EM on capturing different conformational states which greatly contributes to the understanding of the mechanisms of RNA helicases and their regulation. By capturing several conformational states during the helicase cycle functional and regulatory models of the respective enzymes can be reconstituted. Together with the generation of functional mutants the observed regulatory conformations could be validated in both cases.

### Regulation of RNA helicase activity by duplication, self-association or in complex with other protein factors

**Figure 5.**
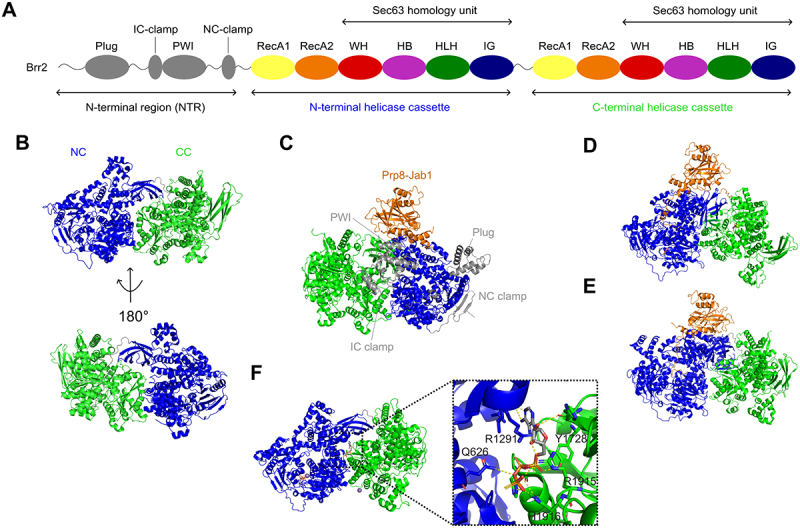
Structural basis of Brr2’s regulation mechanisms. (A) Domain architecture of Brr2. IC-clamp: inter-cassette clamp, NC-clamp: N-terminal cassette clamp, RecA: helicase domain, WH: winged helix domain, HB: helical bundle, HLH: helix-loop-helix, IG: immunoglobulin-like domain. (B) Homo sapiens Brr2 in the apo state (PDB: 4F91). N-terminal (blue) and C-terminal (green) cassette (NC and CC) form an extensive interaction interface. (C) *Saccharomyces cerevisiae* Brr2 in complex with prp-Jab1 (orange) (PDB: 5DCA). The N-terminal region (grey) is forming a broad interaction interface encompassing NC and CC. The plug domain and NC clamp interact with the NC, while IC clamp and PWI extend to CC. (D) *Homo sapiens* (PDB: 4KIT) and (E) *Saccharomyces cerevisiae* Brr2 in complex with prp-Jab1 (PDB: 5M52). Jab1 insert its C-terminal tail into the RNA binding tunnel of the NC. (F) *Chaetomium thermophilum* Brr2 bound to ATPγS (PDB: 6QV4). An additional ATPγS is bound at the interface between NC and CC suggesting another regulatory mechanism.

Another regulatory mechanism of RNA helicases is through oligomeric assemblies either through duplication within on one polypeptide chain or self-association. The well-known Ski2-like helicase Brr2 is a prominent example for an unusual helicase with two helicase cassettes connected in a single polypeptide chain (Figure 5A) [95]. Apart from Brr2, only a few other helicases such as human ASCC3, which is involved in the ribosome-associated quality control (RQT) pathway, exhibit this tandem architecture [96]. Brr2 acts during splicing of pre-mRNA with its crucial role during spliceosome activation. Unlike other spliceosome-associated RNA helicases, which only associate with the spliceosome during the phase in which their activities are required, Brr2 remains associated with the spliceosome after activation [97]. Furthermore, there is evidence that Brr2 is not only required for spliceosome activation but also during catalysis and spliceosome disassembly [98–100]. Therefore, precise regulation of Brr2 during splicing is crucial to ensure correct timing of helicase activation and inhibition. This regulation is achieved by *cis* intramolecular interactions on various different levels and in *trans* by specific protein-protein interactions [97,101]. Despite the high similarity between the two helicase cassettes of Brr2, only the N-terminal cassette (NC) is functional while the C-terminal cassette (CC) exhibits regulatory functions [102]. The NC binds and hydrolyzes ATP and unwinds RNA substrates while the CC acts as a pseudo-enzyme. Due to non-canonical residues at the ATP binding site, the CC can still bind but not hydrolyse ATP [95,102,103]. Despite CC being not enzymatically active, it has been shown to extensively interact with the active NC suggesting a functional link between the two cassettes (Figure 5B) [102]. This is further supported by C-terminal deletion studies, which showed that the CC is required for efficient ATPase and helicase activities of the NC. Notably, blocking of ATP binding in the CC also leads to reduced RNA duplex unwinding of the NC while its ATPase activity is unaffected, suggesting that the two cassettes communicate via long-range, intramolecular interactions [102]. Indeed, the CC can function as an intramolecular cofactor of the NC as it can either enhance or inhibit RNA unwinding of the NC depending on its relative orientation to the NC [104]. Furthermore, the CC acts as a protein-protein interaction platform as it interacts with various different proteins [105]. Given that structural changes in the CC can also affect the NC it is possible that binding of proteins to the CC can regulate NCs activity.

Another unique feature of Brr2, is a long N-terminal region (NTR), which is divided into four sections: the N-terminal ‘plug’ domain consisting of two α-helical hairpins, the intercassette (IC) clamp, the PWI domain and an N-terminal cassette (NC) clamp [106,107]. A crystal structure of yeast Brr2 in complex with the Jab1/MPN1-like domain of Prp8 revealed that the NTR exhibits an extensive interaction site encompassing both N- and C-terminal cassette (Figure 5C). While the ‘plug’ domain and the NC clamp interact with the NC, the IC clamp and PWI extend to the CC, thereby interlocking the two cassettes. Functional analyses of NTR truncations showed that NTR downregulates Brr2^107^. The NTR autoinhibits Brr2 by substrate competition and conformational clamping and thus regulates Brr2 by multiple mechanisms. In the NTR bound state the ‘plug’ domain occupies a position on Brr2s surface which competes with binding of RNA duplex regions during spliceosome activation [97]. The IC clamp further reduces conformational flexibility of the NC required for productive RNA and nucleotide binding by clamping it to the CC. As a result, the ability of the NC to open up between HB and RecA2 and subsequently bind U4 snRNA is impeded. Similarly, the PWI domain interconnects the NC with the CC. Furthermore, the NTR interacts with various splicing factors, which could regulate Brr2’s auto-inhibition via its NTR [107].

A well-known interaction partner of Brr2 is the Jab1/MPN domain of Prp8. Jab1 binds the Sec63 unit of NC and inserts an intrinsically disordered C-terminal tail into the RNA-binding tunnel, thereby inhibiting ssRNA binding and subsequently RNA duplex unwinding [97,101,108]. The same inhibitory binding of Jab1 is observed for human and yeast indicating a conserved regulatory mechanism (Figures 5D, E).

Inhibition by Brr2’s NTR and the Jab1 domain of Prp8 can occur simultaneously suggesting that they complement each other in preventing RNA binding and unwinding [101]. Furthermore, multiple levels of Brr2 autoinhibition that prevent intrinsic ATPase activity in the NC have been identified [109]. In the ATPγS bound state there is no density for the attacking water at the active site suggesting that further conformational changes need to occur upon ATP binding. A possible trigger could be RNA binding to achieve an ATP-hydrolysis-competent state. Such a mechanism would also prevent unproductive ATP hydrolysis in the absence of RNA substrates. U4 snRNA has been shown to activate ATP hydrolysis in the NC. Molecular dynamics (MD) simulations combined with network theory (NWA) and community network analysis (CNA) further suggest that U4 snRNA modulates the conformational dynamics of Brr2 by creating a stronger communication between NC and CC [110].

Interestingly, an additional ATPγS nucleotide is bound at the interface between NC and CC, which suggests another regulatory mechanism (Figure 5F) [109]. As the observed ATPγS is located at the U4/U6 di-snRNA binding site, a nucleotide bound at this position may interfere with RNA binding and may further restrict the conformational flexibility of NC and CC. In fact, high ATP concentrations inhibit U4/U6 unwinding by Brr2 [109].

RNA helicase activity can also be regulated by oligomeric assemblies formed by several RNA helicase molecules. RNA helicases belonging to the superfamilies 3–6 for example are well-known for their hexameric assemblies. Additionally, several DEAD-box helicases, which are typically monomeric, have been observed to self-associate *in vivo*, indicating their potential to oligomerize [111–113]. The *Saccharomyces cerevisiae* DEAD-box helicase Ded1 for example forms trimeric assemblies mediated by its C-terminal region [114]. Interestingly, the three helicases fulfill different functions in the assembly. Two of the protomers, termed ‘loading protomers’ bind single-stranded regions of the RNA. Although, both protomers bind and hydrolyse ATP, they do not contribute to RNA unwinding. Therefore, the third protomer catalyzes the actual strand separation [114]. The different functionality of the individual protomers is a distinct feature of Ded1p. In other oligomerizing helicases it has been shown that each protomer performs all functions and ATP binding and hydrolysis is coordinated between the protomers, which is not observed for Ded1p [114,115]. Interestingly, oligomerization of Ded1p is mediated via its C-terminus. In addition, eIF4G can interfere with Ded1p’s oligomerization by mutually exclusive binding to the C-terminus. The eIF4G-Ded1p complex is still RNA unwinding-competent, albeit with a lower rate compared to the Ded1p trimer [114]. Thus, Ded1p’s helicase activity can be regulated depending on the oligomerization state, which can be altered by eIF4G. Such an oligomerization mechanism has also been suggested for Ded1p’s human ortholog DDX3 [116,117].

A special oligomerization mechanism has evolved in the RIG-I like helicase MDA5. The overall architecture of MDA5 is similar to RIG-I. However, in contrast to RIG-I, which acts as a monomer, MDA5 forms higher-order filamentous oligomers [118]. The formation of these filament structures is crucial for IFN-β signalling and mediates dsRNA-binding cooperativity. Recently published cryo-EM structures of MDA5 bound to dsRNA revealed distinct features of the filament architecture. As it has been shown for RIG-I, the CARDs are not visible in the cryo-EM densities of MDA5 bound to dsRNA, indicating their flexibility and availability for signalling [68,118]. This filamentous oligomerization of MDA5 is mediated by hydrophobic contacts of two interfaces. The first interface is formed between a loop of the Hel1 and the first α-helix of the pincer domain and an adjacent loop of Hel1 of the adjacent MDA5 molecule. The second interface is formed by the C-terminal tail which interacts with the pincer helix of the adjacent MDA5 molecule. Due to the flexibility of the Hel1 loop and the C-terminal tail, the filament assembly is flexible and allows structural versatility necessary for binding t dsRNA. Interestingly, residues forming the interfaces are only conserved within MDA5 and not RIG-I across vertebrates indicating a unique feature of MDA5. Filament formation of MDA5 is crucial for signalling activity, as mutations targeting the filament interfaces abolish IFN-β signalling.

Another regulatory mechanism of RNA helicase activity is by binding of protein cofactors, which interact with the helicase, thereby modulating its activity or function. RNA helicases are often part of multidomain complexes and therefore interact with various other proteins. These interaction partners can either enhance or inhibit helicase activity by inducing structural rearrangements thereby providing additional specificity or regulatory function. Depending on the cellular context, helicase activity can be regulated by binding of a spatially or temporarily specific subset of available protein cofactors. Next to individual cofactors two main evolutionary conserved domains, namely G-patch and middle domain of eukaryotic initiation factor 4 G (MIF4G) domains have evolved and display key roles for modulating helicase activity [30,119]. Most of them act as conformational regulators by inducing structural rearrangements, which either promote or inhibit ATPase and helicase activity.

The MIF4G fold comprises 10 antiparallel α-helices which form five Huntingtin-elongation factor 3-protein phosphatase 2A-TOR1 (HEAT) repeats [120]. A prominent example is the interaction of eIF4A with eIF4G. Binding of eIF4G stabilizes a half-open conformation of eIF4A, which pre-aligns the two RecA domains and facilitates phosphate release. With eIF4G acting as a clamp it reduces the conformational space of eIF4A further facilitating rapid switching between open and closed conformations, thereby accelerating the catalytic activity of eIF4A [121]. Intriguingly, apart from enhancing RNA helicase activity, MIF4G domains can also act as negative regulators of helicase activity. Binding of CWC22 to eIF4A-III for example inhibits its catalytic activity. Despite the interaction interface being similar, it does not stabilize a catalytically favourable conformation and instead induces a misalignment of the conserved motifs involved in ATP- and RNA binding [122].

G-patch proteins are glycine-rich and contain a characteristic G-patch domain (45–50 amino acids) [123]. They interact with DEAH/RHA RNA helicases and enhance their activity. Structural studies of DHX15 in complex with the G-patch protein NKRF revealed that the G-patch motif binds in an extended conformation across the helicase surface thereby restricting the conformational plasticity while maintaining sufficient flexibility for catalysis. This interaction results in an increase in RNA affinity, as well as ATPase and helicase activities [124]. In addition, binding of the G-patch protein Spp2 to Prp2 showed that the key interaction surface is the OB-fold domain which is characteristic of DEAH/RHA RNA helicases. Furthermore, the G-patch adopts a defined fold upon binding along the surface of Prp2, while it is mostly disordered in solution [125]. Interestingly, it has been shown that five different yeast G-patch proteins interact with the OB-fold domain of Prp43 suggesting a mutually exclusive binding mechanism. Competitive binding of these G-patch proteins and their differential local availability thus enables multifunctionality of Prp43 in different RNPs [126]. The interaction of Prp43 with the G-patch protein Pfa1 stabilizes an open conformation, which facilitates phosphate release, thereby accelerating the alternation between open and closed states of Prp43. Consequently, processive translocation along RNA is facilitated [127].

In addition, individual protein cofactors can regulate RNA helicase activity by sterically blocking the access of RNAs to the RNA tunnel or by changing the electrostatic environment, thereby either promoting or inhibiting RNA binding [128–130].

Taken together RNA helicases can multimerize in various assemblies and in complex with other protein cofactors/helicases. However, a common feature of this multimerization is its ability to regulate RNA helicase activity or downstream signalling serving as an additional regulatory mechanism.

## Conclusion and future perspective

Different mechanisms have evolved that regulate RNA helicase activity in *cis* and in *trans*. While the helicase core is conserved in all RNA helicases, they mostly function only with N- and C-terminal auxiliary domains. Since N- and C-terminal extensions are not conserved among RNA helicases, understanding their specific contributions to helicase activity at the molecular level displays a potential target site for small molecule inhibitor discovery. Specific targeting of the unique auxiliary domains and the regulation mediated by them will enable selective modulation of the helicase activity of a selected RNA helicase in human cells while maintaining cellular homoeostasis with minimal side effects at the organismic level. In this regard, cryo-EM displays a valuable tool to capture RNA helicases in various conformational states during the helicase cycle, thereby contributing to the understanding of how the auxiliary N- or C- terminal domains rearrange with respect to the core helicase domains. Therefore, future research on the mechanistic roles of unique auxiliary domains in RNA helicases at the molecular level will be of particular interest. Moreover, understanding the function and regulation of RNA helicases in other organisms such as pathogens and viruses may contribute to find common features in eukaryotic RNA helicases.

## Data Availability

Data sharing not applicable – no new data generated
